# Protocol for quantifying xenografted human cancer cells in zebrafish larvae using Cellpose

**DOI:** 10.1016/j.xpro.2024.103479

**Published:** 2024-12-09

**Authors:** Karina Köpke, Carla-Sophie Lembke, Nynke Oosterhof, Emma S.C. Dijkstra, Judith T.M.L. Paridaen

**Affiliations:** 1 European Research Institute for the Biology of Ageing, University Medical Center Groningen, Antonius Deusinglaan 1, 9713 AV Groningen, the Netherlands

**Keywords:** Cancer, Model Organisms, Computer sciences

## Abstract

Here, we present a protocol for quantifying xenografted human glioblastoma stem cells (GSCs) in zebrafish larvae. We first describe steps for orthotopic xenotransplantation of GSCs into the midbrain of zebrafish larvae, immunofluorescent labeling, and confocal imaging. We then detail procedures for GSC quantification using the Cellpose algorithm. This protocol provides a technique for the semi-automatic segmentation and quantification of human cancer cells in xenograft experiments. With this approach, cancer cell survival and proliferation can be determined in an unbiased manner.

## Highlights

• Orthotopic xenograft of human glioblastoma cells into live zebrafish larvae
• Steps for whole-mount immunostaining and confocal imaging of zebrafish larvae
• Instructions for quantification of xenografted cells using the Cellpose algorithm

## Abstract (editor highlights)

Publisher’s note: Undertaking any experimental protocol requires adherence to local institutional guidelines for laboratory safety and ethics.

## Graphical abstract

**Figure:**
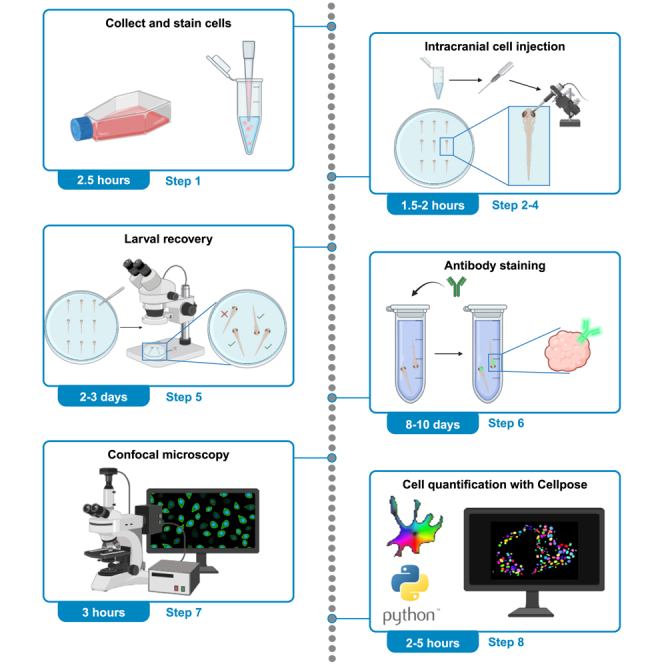

## Before you begin

The protocol describes the injection of human glioblastoma stem cells (GSCs) into the midbrain of live zebrafish larvae, followed by the quantification of GSCs. This protocol can be adapted and applied to inject various human cancer cell lines and patient-derived xenografts into different locations within zebrafish larvae. To locate the cells, staining with CellTracker dye (for example, CMTPX) or the expression of a fluorescent protein is required. Furthermore, for quantification, a human cell-specific antibody is required. Here, we describe the injection of a patient-derived GSC line stained with CMTPX red and anti-human nucleoli antibody.

Cancer (stem) cells can be commercially purchased (e.g., atcc.org), obtained from colleagues or isolated directly from patients as described previously.^1^

### Institutional permissions

All experiments on zebrafish larvae (below 120 h post-fertilization (hpf)) were approved by the Animal Welfare Body of the University of Groningen (University Medical Center Groningen). Housing of the zebrafish strains that generated the larvae took place in the Central Animal Facilities UMCG (Facility accreditation of the Dutch authorities under license number NVWA/2024/010040933).

In case human cells are isolated directly from tumor tissue, approval by the Institutional Ethical Review Board is required. The isolation of tumor tissue is not described in this protocol but can be found in multiple other publications.^2^^,^^3^

Use of zebrafish larvae beyond 120 hpf should be carried out in accordance with institutional and national guidelines and legislation on use of experimental animals. Obtain the necessary permissions and licenses from the appropriate animal experimentation committee(s) prior to starting this protocol.

### Culturing of glioblastoma stem cells

**Timing: 2–3 weeks**

Here, the cell culture protocol for glioblastoma stem cells GG16^3^ or GCS23^4^ (growing non-adherent) is described. Protocols can vary from cell type to cell type and may have to be adapted depending on the cell line of choice.

1. Seed glioblastoma stem cells (GSC) into a T25 flask:
  a. Thaw a cryogenic vial with 1 × 10^6^ cells/mL in a 37°C water bath until the medium is almost completely thawed.
  b. Directly add the cell suspension dropwise into 5 mL of NeuroBasal medium (NBM) in a 15 mL falcon tube using a P1000 pipet.
  c. Centrifuge cells at 100 × *g* for 4 min at 20°C without breaking.
  d. Discard the supernatant and resuspend the cells in 4 mL NBM with supplements (NBM+).
  e. Seed the solution into T25 flasks and incubate at 37°C with 5% CO2.

***Note:*** Pre-warm NBM+ and PBS in a water bath at 37°C and Accutase at 20°C and sterilize the cell culture hood as well as the equipment that is used inside of the hood, such as pipettes. Cryogenic vials of GSCs usually contain 1 × 10^6^ cells in NBM with supplements and 20% v/v cell-culture grade DMSO. As DMSO is toxic for the cells, ensure rapid conduction of step 1b. It is recommended to not seed more than 1 × 10^6^ cells per T25 flask. The first passage should follow according to the culture protocol described below.

**Figure 1:**
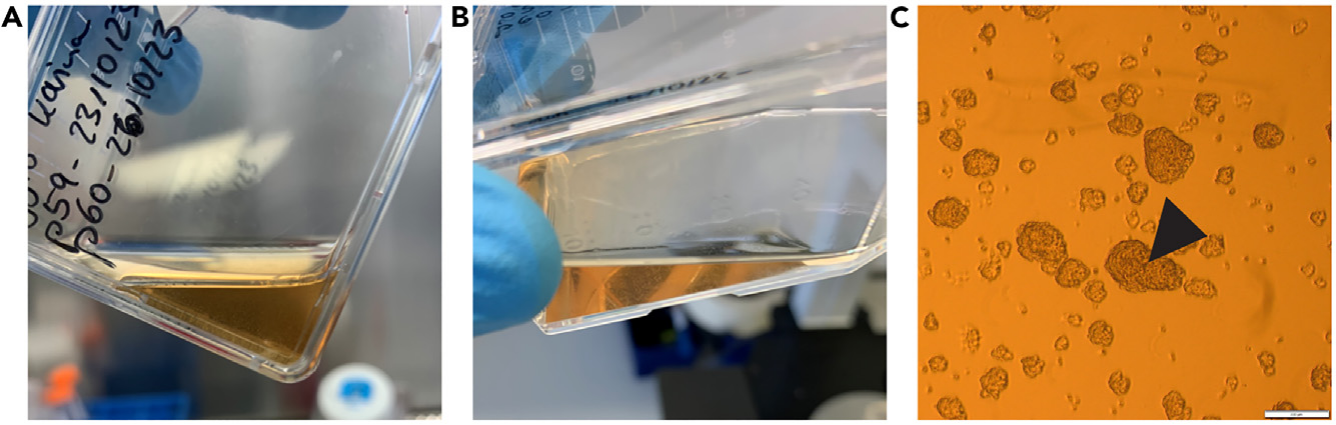
Culturing of Glioblastoma stem cells (A and B) show GG16 cell spheres in suspension right before passaging. Note that spheres are visible by eye and that the color of the medium is turning orange. (C) shows microscopic image of GG16 spheres at 4x magnification before passaging. The arrowhead indicates a blackish center of a sphere. Scale bar = 200 μm.
  b. Transfer cells to a 15 mL falcon tube and pellet cells by centrifuging at 100 × *g* for 4 min at 20°C without breaking.
  c. Aspirate supernatant and resuspend the cells in 2 mL of pre-warmed cell culture-grade PBS.
    **CRITICAL:** Be careful not to touch the cells with the pipette (tip) directly. Recommended for use are 2 mL or 5 mL glass pipettes. The pipette controller should be at minimum speed.
  d. Repeat cell pelleting by centrifuging at 100 × *g* for 4 min at 20°C without breaking.
  e. Aspirate the supernatant and resuspend cells in 400 μL of Accutase pre-warmed to room temperature to accomplish a single cell solution. For this, repeatedly pipette the cells up and down and ensure the pellet is fully resuspended.
  f. Incubate for 5 min at 20°C.
    ***Note:*** In case the cells are still in spheres or sedimenting after the Accutase-incubation time is over, flicking the tube can help loosen them up. Otherwise, resuspension with a pipette tip can help. It is important to note that the cells automatically sink down to the bottom of the tube, so it may look like there is still a pellet (Figure 2A). By gentle shaking or flicking the tube, it can be ensured that the cells are distributed into the suspension again and no spheres are visible anymore (Figure 2B).

2. Passage GSCs into a T25 flask:
  a. Cells should be passaged when the color of the culture medium is turning orange or when around 10% of the spheres show a blackish center (Figure 1C) (Figures 1A and 1B). For GSC23 and GG16 cell lines, cells are passaged every 3–4 days.

**Figure 2:**
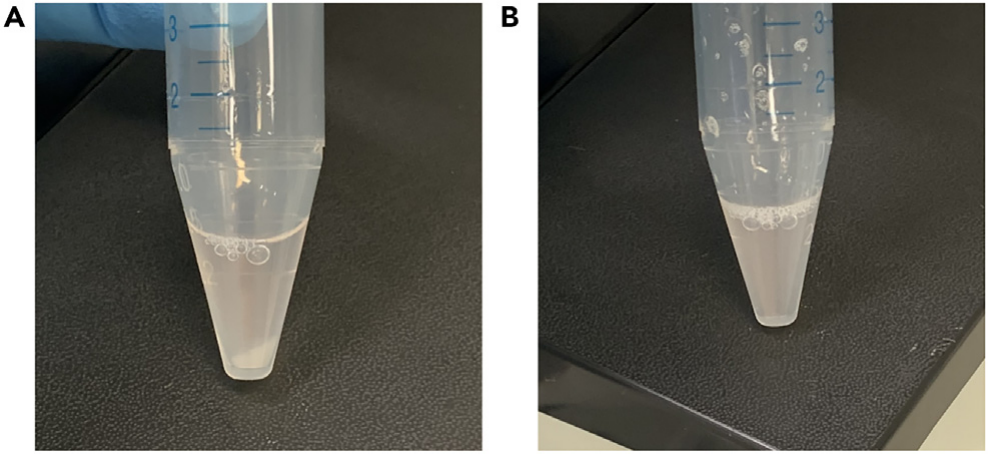
Sphere dissociation using Accutase (A) Shows cells accumulating at the bottom of the tube after incubation with Accutase. (B) Shows resuspension of the cells in Accutase after gentle flicking of the tube.
  g. Add 1.6 mL of NBM+ pre-warmed to 37°C and ensure suspension by pipetting up and down. Confirm by visual inspection that the spheres are dissociated.
  h. Count cells using a Neubauer chamber (Hemocytometer).
  i. Add to a new T25 flask 4 mL of NBM+ pre-warmed to 37°C.
  j. Transfer cells to new T25 flask at a 1.75–2.5 × 10^5^ cells/mL dilution. Here, this is equal to around 700,000 to 1 million cells in 4 mL medium.
  k. Maintain cells at 37°C and 5% CO_2_ until around 10% of the spheres show a blackish center.
    ***Note:*** If the total number of cells needed for an experiment is more than one T25 flasks yields, cells can be split into multiple flasks. For this, execute 2i–j for multiple flasks.
    ***Note:*** Passage every 3–4 days, once medium turns orange and spheres become visible by eye. In case the spheres are not yet visible by eye when inspecting the cells on the day of passage, fresh medium can be added. If spheres are sticking together, attached to the bottom of the flask, show more than ∼20% blackish centers or the medium has turned yellow, the cells are beyond passaging and should not be used anymore. Passage cells at least 3 times before using them in experiments, and do not exceed a passage number of 65.

### Pull injection needles

**Timing: 10 min**

3. Using thin wall capillary and the micropipette puller, pull the injection needles.
  a. Perform a ramp test to determine the melting temperature of the glass capillaries according to the manufacturer’s instructions (https://www.sutter.com/manuals/P-1000_QuickStartGuide.pdf).
  b. Use the following (unitless) parameter values to pull the injection needles: Heat Ramp test value + 10, Pull 75, Velocity 70, Delay 100, Pressure 200.

**Figure 3:**
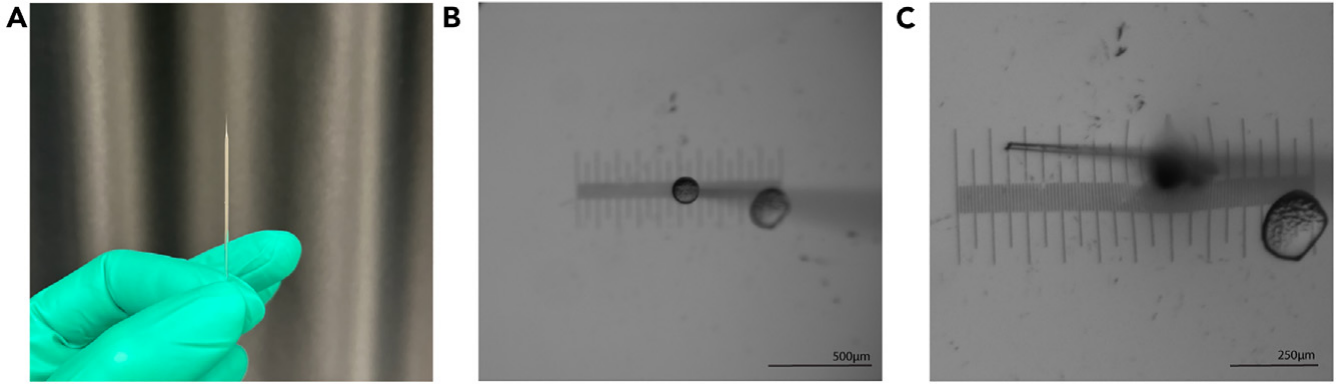
Injection needle preparation (A) Shows correctly pulled and closed injection needle. (B) Shows ejection volume of roughly 500 pL in mineral oil, with micrometer stage in the background. (C) Shows the approximate size of an opened needle tip with micrometer stage in background. Scale bar = 500 μm (B) and 250 μm (C).

***Note:***Figure 3A shows a properly pulled injection needle. This step can be done at any time before the experiment. Pulled needles can be stored in a 10 cm petri dish using double sided tape. Consult your micropipette puller manual to make desired adjustments to increase or decrease the taper of the injection needles.

### Prepare software and computing environment

**Timing: 2 h**

This section describes the software and packages that are required to execute this protocol and how to set them up. This protocol works on Windows, as well as Mac operating systems, even though we describe the Windows-based protocol below. Ensure that your operating system supports Python (version 3.7.8.) installation and use. The bioinformatics pipeline is based on the previously published “Cell counter manual”, where a manual for installation on Mac operation systems is provided as well.^5^

**Figure 4:**
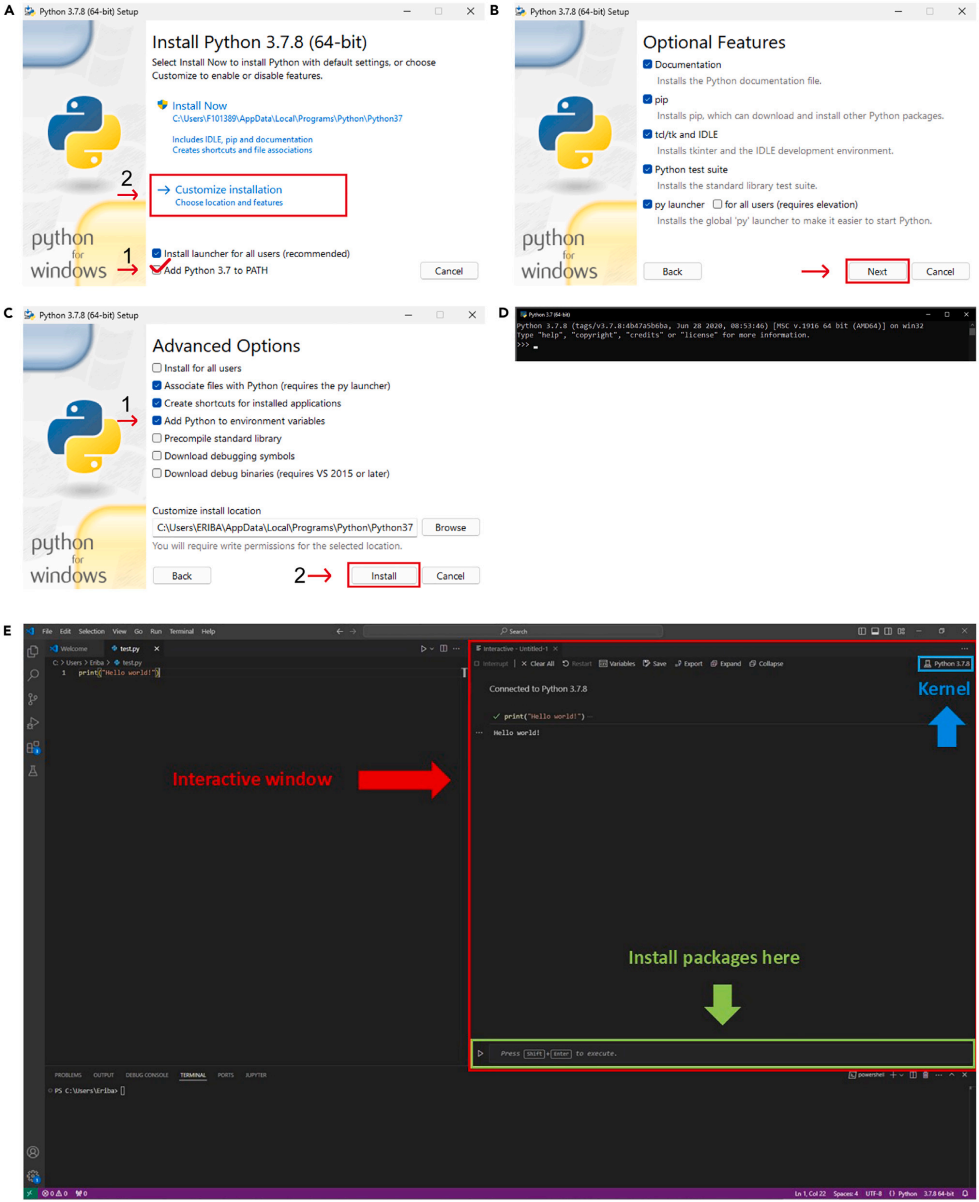
Prepare software and computing environment (A–C) show the steps for installing python. (D) Shows the window that appears when python is installed correctly. (E) Shows an overview of the Integrated Development Environment Visual Studio Code with indications for the interactive window, the kernel and where to install packages.
  c. To make sure Python has been installed, type **python** in the terminal and press ‘enter’. Python will be started in the terminal (Figure 4D) and can be closed by typing **exit()** and pressing “enter”.
  d. Create a new folder from which the analysis will be run.
    ***Note:*** It is recommended to create this folder in “Documents” for simplicity and traceability and name it e.g. python_scripts.
    ***Note:*** When naming files or folders, it is useful to replace empty spaces between words with an underscore as this can help avoid possible access problems later.
  e. Copy all the **.py** files necessary for the cell counting to this folder. You can find these files on GitHub (https://github.com/nynkeoosterhof1/xenograft-quantification).
  f. Start VSCode and choose “open folder” on the starting screen and select the folder that was just created and includes the python scripts.
  g. Install the Jupyter extension by pressing Ctrl+Shift+X to open the extension panel. Within the panel, search for Jupyter and follow the instructions to install the extension.
  h. It is recommended to create a virtual Python environment to be able to keep projects run in VSCode separate by using the following command in the terminal:
    > python -m venv <venv_name>
    ***Note:*** If using a virtual environment, make sure to select and activate it before proceeding to any package installations by using the select interpreter command from the Command Palette, which can be accessed with Ctrl+Shift+P. The selected interpreter will be displayed in the status bar.
    ***Note:*** <venv_name> should be replaced by the name of the virtual environment, e.g. venv_cellcount. Further, VScode may ask whether this new virtual environment should be used for this folder. If so, click **yes**.
  i. Install the *ipykernel* package by creating a new file in the VS explorer and call it **test.py**. Within this file type.
    > print("Hello World!")
    ***Note:*** Right-click on the file in the explorer and choose to run the file in an interactive window. This opens a pop-up window asking to install the *ipykernel* package. Install this package.
  j. Install the required packages for using the zebrafish xenograft cell counter by running the following commands in the interactive window.
    i. Run the code by either pressing the **“play”** button or pressing **shift+enter**.
      > pip install scikit-image==0.19.3
      > pip install cellpose==2.1.0
      > pip install numpy==1.21.6
      > pip install pandas==1.3.5
      > pip install matplotlib-inline==0.1.6
      > pip install read_lif==0.4.0
      > pip install openpyxl==3.1.3
      ***Note:*** The version number is indicated following the two equal signs.
      ***Note:*** Ensure that when using the interactive window for this, the kernel in use is the one associated with the created virtual environment. The name of the kernel is displayed in the upper right corner of the interactive window and should have the same name as the virtual environment that was created. To change the kernel, click on it and select the desired kernel (Figure 4E).

4. Set up the computing environment on the computer by installing python, Visual Studio Code and required packages:
  a. Install Python and an IDE (Integrated Development Environment) that allows you to write and run Python code. Examples of IDE’s that can be used are Visual Studio Code (VSCode, explained in this manual) and Spyder.
    i. Python: https://www.python.org/downloads/
    ii. VScode: https://code.visualstudio.com/docs/python/python-tutorial.
    iii. Instructions for VScode set up: https://code.visualstudio.com/docs/python/python-tutorial.
      ***Note:*** If Cellpose will not be run on a computer cluster, make sure to install Python 3.7. Later versions may interfere with the package version used in this protocol. Later Python 3.7 versions may work as well but have not been tested in this publication.
  b. To install that Python version, click on Python 3.7.8, scroll down and download the **Windows x86–64 executable installer**.
    ***Note:*** When executing the installer to install Python, make sure to tick the box saying ‘**Add Python to environment variables**’ in the advanced options (Figures 4A–4C). This will make it easier to follow the next step of the manual. To check if Python is correctly added as an environment variable, open the windows command line and simply type **python**, which should open python (Figure 4D).

## Key resources table

**Table:** 

REAGENT or RESOURCE	SOURCE	IDENTIFIER
**Antibodies**		

Anti-hs nucleoli (1:100)	Abcam	Cat# 190710
Alexa 488 or Alexa 555 (1:500)	Thermo Fisher Scientific	Cat# A-10680 or A-21422

**Chemicals, peptides, and recombinant proteins**		

Accutase	Thermo Fisher Scientific	Cat# A1110501
Agarose, low melting point	MP Biomedicals	Cat# AGAL0050
Animal-free recombinant human EGF	PreproTech	Cat# AF-100-15
B27 supplement	Thermo Fisher Scientific	Cat# 17504044
Bovine serum albumin	Sigma-Aldrich	Cat# A9647-100G
Calcium chloride (CaCl2)	Fisher Scientific UK	Cat# 10043-52-4
CellTracker Red CMTPX dye	Thermo Fisher Scientific	Cat# C34552
DAPI	Roche	Cat# 28718-90-3
DMSO	Sigma-Aldrich	Cat# D2438
GlutaMAX	Thermo Fisher Scientific	Cat# 35050061
Hydrogen chloride (HCl)	Sigma-Aldrich	Cat# 7647-01-0
Magnesium sulfate (MgSO4)	Sigma-Aldrich	Cat# 7487-88-9
Methylene blue	Sigma-Aldrich	Cat# 50484
Mineral oil	Sigma-Aldrich	Cat# 8042-47-5
Neurobasal medium A	Thermo Fisher Scientific	Cat# 21103049
Paraformaldehyde powder	Sigma-Aldrich	Cat# 30525-89-4
Penicillin/streptomycin	Thermo Fisher Scientific	Cat# 15140122
Phosphate-buffered saline (PBS)	Thermo Fisher Scientific	Cat# 14040133
Polyvinylpyrrolidone	Sigma-Aldrich	Cat# 856568
Potassium chloride (KCl)	Sigma-Aldrich	Cat# 7447-40-7
Proteinase K	Roche	Cat# 03450384103
Recombinant human FGF-basic	PreproTech	Cat# 100-18B
Sodium chloride (NaCl)	Sigma-Aldrich	Cat# 7647-14-5
Tricaine (MS222)	Sigma-Aldrich	Cat# 886-86-2
Tris base	Sigma-Aldrich	Cat# TRISRO
Triton X-100	Sigma-Aldrich	Cat# 9036-19-5

**Experimental models: Cell lines**		

Human: GG16 glioblastoma stem cell line	Joseph et al.^3^	N/A
Human: GSC23 glioblastoma stem cell line	Baht et al.^4^	N/A

**Experimental models: Organisms/strains**		

Zebrafish: Casper (Tg(*roy* ^*a9/a9*^*;nac* ^*w2/w2*^); both males and females; 3–18 months; 70–120 hpf	ZIRC	ZIRC catalog ID: ZL1714

**Software and algorithms**		

Cell Counter	Casas-Gimeno et al.^5^	https://github.com/nynkeoosterhof1/cell-counter
Xenograft Quantification	This article	https://github.com/nynkeoosterhof1/xenograft-quantification
Cellpose	Stringer et al.^6^	https://cellpose.org/
Fiji platform	Schindelin et al.^7^	http://imagej.net/Fiji
Leica Application Suite X	Leica Microsystems	RRID:SCR_013673
Python version 3.7.8	Python Software Foundation	https://www.python.org/
Visual Studio Code	Microsoft (open source)	https://code.visualstudio.com/

**Other**		

100 mm non-treated petri dish	Sigma-Aldrich	Cat# P5606-400EA
15 mL Falcon tube	Corning	Cat# 352095
1 mL syringe	Sigma-Aldrich	Cat# Z683531-100EA
BD Microlance 26G needle	Fisher Scientific	Cat# 10703815
Centrifuge	Eppendorf	Centrifuge 5804R
CO_2_ incubator	N/A	N/A
Confocal laser scanning microscope	Leica	Leica TCS SP8 X
Cryogenic vial	Sarstedt	Cat# 72.379
Dumont fine forceps	Fine Science Tools	Cat# 11254-20
Eppendorf Microloader tips	Fisher Scientific	Cat# 10289651
Eppendorf tubes (1.5 and 2 mL)	Sarstedt	Cat# 72695400 (2 mL), Cat# 72706400 (1.5 mL)
Fluorescent stereo microscope	Leica Microsystems	Leica MZ FLIII
Foot switch for PV820	World Precision Instruments	Cat# 3260
Glass bottom dish, 35 mm	MatTek	Cat# P35G-1.5-10-C
Glass Pasteur pipette	Hirschmann	Cat# 9260101
Hemocytometer counting chamber	Sigma-Aldrich	Cat# Z359629
Immersion oil	Sigma-Aldrich	Cat# 56822-50ML
Incubator IMC18 Heratherm 120-230VAC	Thermo Fisher Scientific	Cat# 50125882
Leica metric stage micrometer	VWR	Cat# 15147-890
Magnetic base	World Precision Instruments	Cat# M10
Micromanipulator with 12 mm clamp right-handed	World Precision Instruments	Cat# M3301R
Micropipette puller P1000	Sutter Instrument	Cat# P-1000
Microscope camera	Leica Microsystems	Leica DFC3000 G
Microscope light source	Leica Microsystems	Leica CLS 150 X
Mini block heater	VWR	Cat# 10153-318
Non-filament microcapillaries	World Precision Instruments	Cat# TW100F-4
Pipette tips (P1000, P200, P20, P2)	N/A	N/A
Pneumatic PicoPump PV820	World Precision Instruments	Cat# SYS-PV820
Polystyrene round bottom tube	Corning	Cat# 352235
Sample rocker	N/A	N/A
Steel base plate	World Precision Instruments	Cat# 5052
Stripette serological pipets (2, 5, 10, and 25 mL)	Corning	Cat# 4486 to 4489
T25 cell culture flasks	Sarstedt	Cat# 83.3910.302
Transfer pipette	Sarstedt	Cat# 86.1174.300
Water bath	N/A	N/A

## Materials and equipment

### Recipes for solutions used in this protocol

#### Short recipes

• 10 mM CMTPX dye solution: Mix 50 μg of CMTPX dye with 7.3 μL DMSO. Aliquot dye solution and avoid repeated freeze/thaw cycles and excessive light exposure. Store at −20°C in darkness.
• 2% Polyvinylpyrrolidone solution: Mix 200 mg of Polyvinylpyrrolidone (PVP) powder with 10 mL of NBM. Store at 4°C for up to 4 weeks.
• 20% Bovine Serum Albumin stock: Mix 2 g of bovine serum albumin (BSA) powder with 10 mL 1x PBS. Aliquot in 1.5 mL Eppendorf tubes and store at −20°C for up to a year.
• DAPI solution: Make a stock solution of DAPI in 1x PBS with a concentration of 1 mg/mL. Store at −20°C in darkness.

### Recipes with ≥3 ingredients

**Table:** Neurobasal medium + supplements (NBM+)

Reagent	Final concentration	Amount
B27 supplement	2%	2 mL
GlutaMAX	1%	1 mL
Penicillin/Streptomycin	1%	1 mL
Animal-Free Recombinant Human EGF	20 ng/mL	40 μL
Recombinant Human FGF-basic	20 ng/mL	20 μL
Neurobasal Medium (NBM)	N/A	96 mL
**Total**	**N/A**	**100 mL**

***Note:*** Store at 4°C for up to 4 weeks. Avoid repeated freeze thaw cycles for B27, EGF, and FGF by aliquoting and storing at −20°C.

**Table:** 0.86 M Tris buffer, pH 9.5

Reagent	Final concentration	Amount
Tris Base	0.86 M	104.4 g
dH20	–	Bring to 1000 mL
HCl	N/A	N/A
NaCl	N/A	N/A
**Total**	**N/A**	**1000 mL**

***Note:*** Add Tris base to 800 mL dH_2_O and adjust the pH to 9.5 using HCl and NaCl. Adjust volume to 1000 mL using dH_2_O. Store at 20°C for up to a year.

**Table:** 4% Paraformaldehyde solution

Reagent	Final concentration	Amount
Paraformaldehyde powder	4%	40 g
1x PBS	N/A	add up to 1000 mL
HCl	0.1 M	N/A
NaOH	1 M	N/A
**Total**	**N/A**	**1000 mL**

***Note:*** Preheat 800 mL of 1x PBS to 60°C. Add 40 g of paraformaldehyde powder and stir until the solution is clear. Allow the solution to cool and subsequently filter it. Adjust the volume of the solution to 1000 mL with 1x PBS. Recheck the pH and adjust it with small amounts of HCl/NaOH to approximately 6.9 pH.

Aliquot the solution to 10 mL and store at −20°C for up to a year, avoiding repeated freeze/thaw cycles.

**CRITICAL:** Paraformaldehyde is a toxic carcinogen and irritant. Use a fume hood and personal protection equipment and work carefully.

***Note:*** If desired, premade 4% PFA solution can be purchased online, e.g. at https://www.emsdiasum.com/formaldehyde-aqueous-solution-paraformaldehyde-aqueous-solution-em-grade.

**Table:** 10x Tricaine solution, pH 7

Reagent	Final concentration	Amount
Tricaine (MS-222)	0.16%	1.6 g
Tris Buffer	0.86 M	N/A
dH2O	N/A	add up to 1000 mL
**Total**	**N/A**	**1000 mL**

***Note:*** Add 1.6 g Tricaine to 800 mL dH_2_O and adjust the pH to 7 using Tris solution (0.86M, pH 9.5). Adjust volume to 1000 mL using dH_2_O. Aliquot the solution to 10 mL and store at −20°C for up to a year, avoiding repeated freeze/thaw cycles.

**Table:** 60x E3 stock solution

Reagent	Final concentration	Amount
Sodium Chloride (NaCl)	300 mM	17.4 g
Potassium Chloride (KCl)	100 mM	0.76 g
Calcium chloride (CaCl2)	200 mM	2.92 g
Magnesium sulfate (MgSO4)	2 mM	0.49 g
dH20	N/A	Bring up to 1000 mL
**Total**	**N/A**	**1000 mL**

***Note:*** Store at 20°C for up to 12 months.

**Table:** 1x E3 working solution

Reagent	Final concentration	Amount
60x E3 stock solution (store at 4°C)	1x	16.7 mL
Methylene Blue	0.05%	75 μL
dH20	N/A	bring up to 1000 mL
**Total**	**N/A**	**1000 mL**

***Note:*** Store at 20°C for up to 12 months.

## Step-by-step method details

### Stain cells with fluorescent dye

**Timing: 2.5 h**

This step outlines the protocol of staining non-adherent GSCs with CellTracker Red CMTPX dye. Staining is recommended to validate the staining with anti-human nucleoli antibody. Other CellTracker dyes may be used as well. All the work needs to be done in a sterile environment.

1. Prepare a 10 mM cell dye solution for CMTPX in DMSO.

***Note:*** It is recommended to aliquot the dye at 1 μL at −20°C once the dye solution has been prepared to prevent multiple freeze/thaw cycles as this can impact the quality of the dye.

2. Prepare a 2% polyvinylpyrrolidone solution in NBM.
3. Passage the cells as described in the “Culturing of glioblastoma stem cells (GSCs)” section.
  a. Pool all cells that remain after passaging the cells into a 15 mL falcon tube. It is advised to use all cells for staining to ensure a sufficient number of cells for injection.
  b. Centrifuge cells at 100 × *g* for 4 min at 20°C without breaking.
  c. Remove the supernatant and resuspend the cell pellet in 0.5 μL CMTPX (5 μM) dissolved in 999.5 μL NBM without supplements pre-warmed to 37°C and incubate for 30 min at 37°C.
4. Stain the non-adherent cells.
  **CRITICAL:** Reduce the time the dye is exposed to light. Light exposure can be prevented by wrapping the tube containing the dye in aluminum foil.
  a. After incubation, suspend the cells in 10 mL NBM pre-warmed to 37°C and centrifuge at 100 × *g* for 4 min at 20°C without breaking.
  b. Remove the supernatant and resuspend the cells in 1 mL 2% polyvinylpyrrolidone (PVP) solution.
  c. Transfer the suspension to a 1.5 mL eppendorf tube.
  d. Filter the suspension through a 5 mL polystyrene round-bottom tube with cell-strainer cap using a 1–5 mL syringe and 26G needle.
    **CRITICAL:** Be careful not to use too much force to avoid overflow of the suspension outside of the filter cap tube.
    ***Note:*** For filtering the suspension through the filter cap tube, the cap can be lifted a bit from the tube to enable passing through the filter mesh. The tube listed here can be replaced by similar tubes, as long as the mesh size is 35 μm.
5. Count cells using the hemocytometer.
6. Centrifuge cells at 100 × *g* for 4 min at 20°C without breaking and remove the supernatant.
7. To get a concentration of 0.25 × 10^6^ cells/μL for the xenograft, suspend the cells in 2% PVP depending on the number of cells counted. Other tumor cell types may require different cell concentrations for xenograft.^8^

***Alternatives:*** The steps above are described for non-transgenic cell lines. If a cell line expressing a fluorescent marker is available, this can also be used. For this, passage cells as usual and start this protocol at step 5.

### Prepare and mount larvae for injection

**Timing: 45 min**

**Figure 5:**
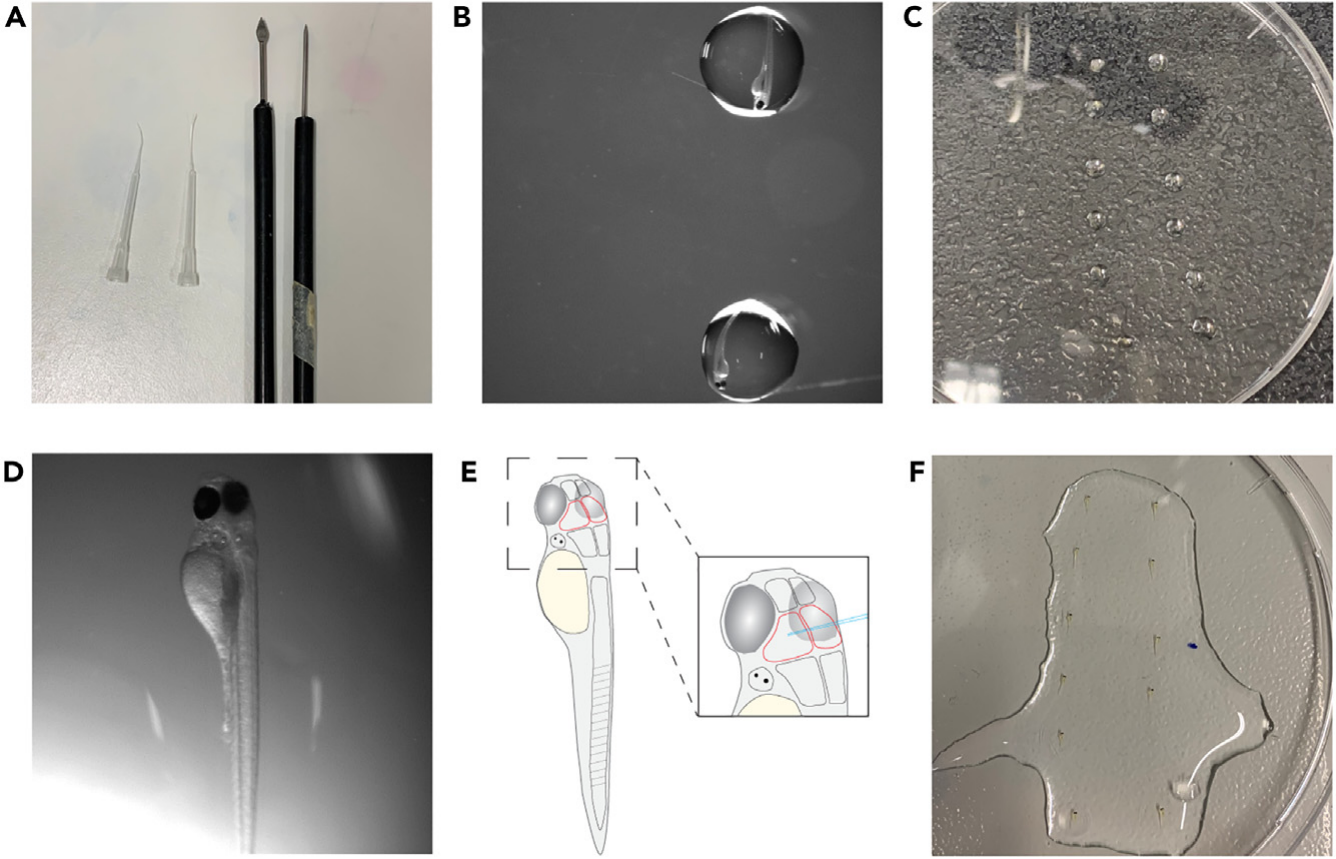
Prepare and mount larvae for injection (A) Shows utensils used for positioning and later removal of agarose. (B) Shows larvae placed in a drop of medium in the lid of a 10 cm Petri dish. (C) Shows an overview of larvae in drops of medium. Larvae are lined up from top to bottom and in rows to later facilitate the injection procedure. (D) Shows a larva positioned in agarose. Note the slight tilt of the body to the right lateral side. (E) Shows a schematic of a positioned larva with the midbrain outlined in red as well as the brain enlarged to show correct needle positioning. (F) Shows agarose-embedded larvae with added E3 and 1x tricaine solution on top.

Here, the steps of larva selection for injection, as well as positioning the larvae for orthotopic xenograft are described (see also Figure 5).

8. Collect larvae at 70–75 hpf^9^ and select healthy larvae (Figure 5D) for xenotransplantation.
  a. Use a stereomicroscope to remove any dead, mis-/underdeveloped and abnormal larvae.
9. Anesthetize the larvae for injections.
  a. To ensure minimal discomfort and movement of the larvae, transfer the larvae to a 10 cm Petri dish with 1x E3 containing 1x Tricaine solution.
10. Mount the larvae into the injection position.
  a. Transfer a 1.5 mL tube of 1.5% low-melting point agarose in E3 to a heating block at 60°C.
  b. Transfer single larvae into the lid of a clean 10 cm petri dish (Figures 5B and 5C) using a plastic transfer pipet.
  c. With a plastic transfer pipet, remove excess E3 medium so that the larva is still minimally submerged in medium.
  d. Using a plastic transfer pipet, add a drop of 1.5% low-melting point agarose on top of the larva (Figures 5C and 5D).
    ***Caution:*** Make sure the temperature of the agarose does not exceed 45°C, as otherwise the health of the larvae can be impacted. This can be done by touching the transfer pipet filled with agarose. Be careful and try to avoid diluting the agarose by adding too much E3 medium.
  e. Position the larva rapidly, making use of a blunt surgical/dissection instrument to orient the larva (e.g., using microloader pipette tips with shortened ends, Figure 5A).
    ***Note:*** Watching through the microscope, use the positioning tool to turn and position the larva inside of the agarose. Aim to position the larva in a slight lateral tilt, easiest to recognize with the position of the eyes (Figures 5D and 5E). For injections into the head/midbrain, it is helpful to have one of the pipette tips positioned on the ventral side of the larvae head to lift it up towards the surface of the agarose. This will reduce the distance the injection needle is passing through agarose and facilitate the injection procedure. If necessary, hold the larva in position until the agarose is solidified.
  f. Repeat step 10c–e for all larvae needed.
    ***Note:*** A 10 cm petri dish can fit 10–15 drops of 1.5% low-melting point agarose containing larvae (Figures 5C and 5F). During placement of the drops, be cautious to not place the drops too close to each other. If needed, multiple petri dishes can be used. It is recommended to position all larvae in the same direction to facilitate later injection.
  g. Let the agarose solidify for 5–10 min and add 1x E3 medium with 1x Tricaine solution to prevent drying out the agarose, as this will detach the drops from the dish (Figure 5F).

### Set up microinjector

**Timing: 15 min**

This step describes setting up a Pneumatic picopump (PV820, World Precision Instruments) equipped with foot pedal and micromanipulator, which are coupled to a Leica M80 stereomicroscope with LED base (TL3000) for zebrafish micro-injections and is broadly applicable also to other microinjectors. Consult the manual of the microinjector for more details on how to adjust injection parameters.

***Note:*** Adjust the settings in a way that there is no cell suspension leakage from the needle and no extra fluid/tissue is being taken up after injection. It is important that the pressure is still high enough to actually eject the cells. To achieve this, it is an interplay of setting the hold and eject pressure at the right settings. While this can be different for other setups, we will describe the settings for our setup.

11. Set up the microinjector.
  a. Turn on the microinjector and connect it to compressed air.
  b. Adjust the compressed air influx so that the eject pressure increases to 20 psi.
  c. Set the eject pressure between 20 and 40 psi.
    ***Note:*** It is recommended to start at 20 psi, as otherwise the ejection pressure might be too high and destroy vital parts of the zebrafish larva (brain).
  d. Set the hold pressure below 1 psi.
    ***Note:*** The hold pressure is very sensitive, never turn it to 0 psi, and too high hold pressure will lead to leaking of the cell suspension from the needle.
  e. Set the time range for injection to 100 ms and set the time to 1. With this setting, the duration of the pressure ejecting the cells can be modified to adjust the volume of the ejected solution.
    **CRITICAL:** Make sure to set the switch to a timed output, as when on the gated setting the efflux will be of the time the ejection pedal is pressed and not the set time.
  f. Load the glass capillary needle with 2–4 μL cell suspension using a 20 μL microloader pipette tip (Video S1).
    ***Note:*** Ensure resuspension of the cells by flicking the eppendorf tube prior to pipetting up the cells into the microloader tip, as the cells will naturally start to form a loose pellet in the bottom of the tube.
  g. Attach the needle to the microinjector.
    ***Note:*** Make sure to insert the capillary end of the needle up to the top of the probe and to tighten the screw carefully to not break the injection needle.
  h. Calibrate the needle to adjust the ejection volume to roughly 500 pL; in this case the volume contains roughly 125 cells based on the cell concentration in the injection solution (Figure 3B).
    ***Note:*** Place a drop of mineral oil on the stage micrometer. Using Dumont forceps, cut the needle tip open by snipping off the needle end.
    **CRITICAL:** Start with a small opening that can be increased later. The optimal diameter of the opening depends on the cell type and size. In general, the needle tip opening should be wide enough to ensure flow of individual cells but small enough to not eject multiple cells at once and to not damage the larva (Figure 3C).
  i. Move the needle tip into the mineral oil droplet and start pressing the ejection pedal to eject the cell solution.
    ***Note:*** In the tip of the needle, air might accumulate, so to circumvent having to press the pedal multiple times, one can very carefully switch to the gated output on the picopump and briefly press the pedal. Usually, with stereomicroscopes, the individual cells can be discerned within the needle and the setting can then be returned to timed output.
  j. According to the cell concentration in the solution and the desired number of cells to inject, increase the ejection volume.
    ***Note:*** To increase the injection volume do either of the following:
    Cut more of the needle tip off. This will yield a considerable increase in volume, so it should be used when the ejection volume is clearly too little, or cells cannot easily pass through the needle opening.
    For more precise fine tuning of the volume, it is recommended to increase ejection time or the ejection pressure carefully. In case the ejection volume is too high, the ejection time or eject pressure can be carefully decreased.
    ***Note:*** It is easier to increase the ejection volume than to decrease it, which is why it is recommended to start with a lower injection volume and increase it until the desired volume is reached. If the ejection volume is too high and cannot be further decreased by adjusting the ejection volume or pressure, we recommend starting with a new needle from Step 11f onwards. It is not recommended to use injection volumes of above 1 nL, as this usually leads to cells being injected into the ventricle or injecting too much liquid into the brain, leading to increases in larval mortality.

Video S1. Loading of injection needle, related to step 11f The movie shows the loading of the needle with cell solution using a microloader tip.

### Intracranial cell injection

**Timing: 30–45 min**

This step describes the injection of cells into the midbrain of the zebrafish larva. It is important to proceed with care to not affect the larval structures and influence the larvae survival. The timing is estimated for 10–15 larvae.

**Figure 6:**
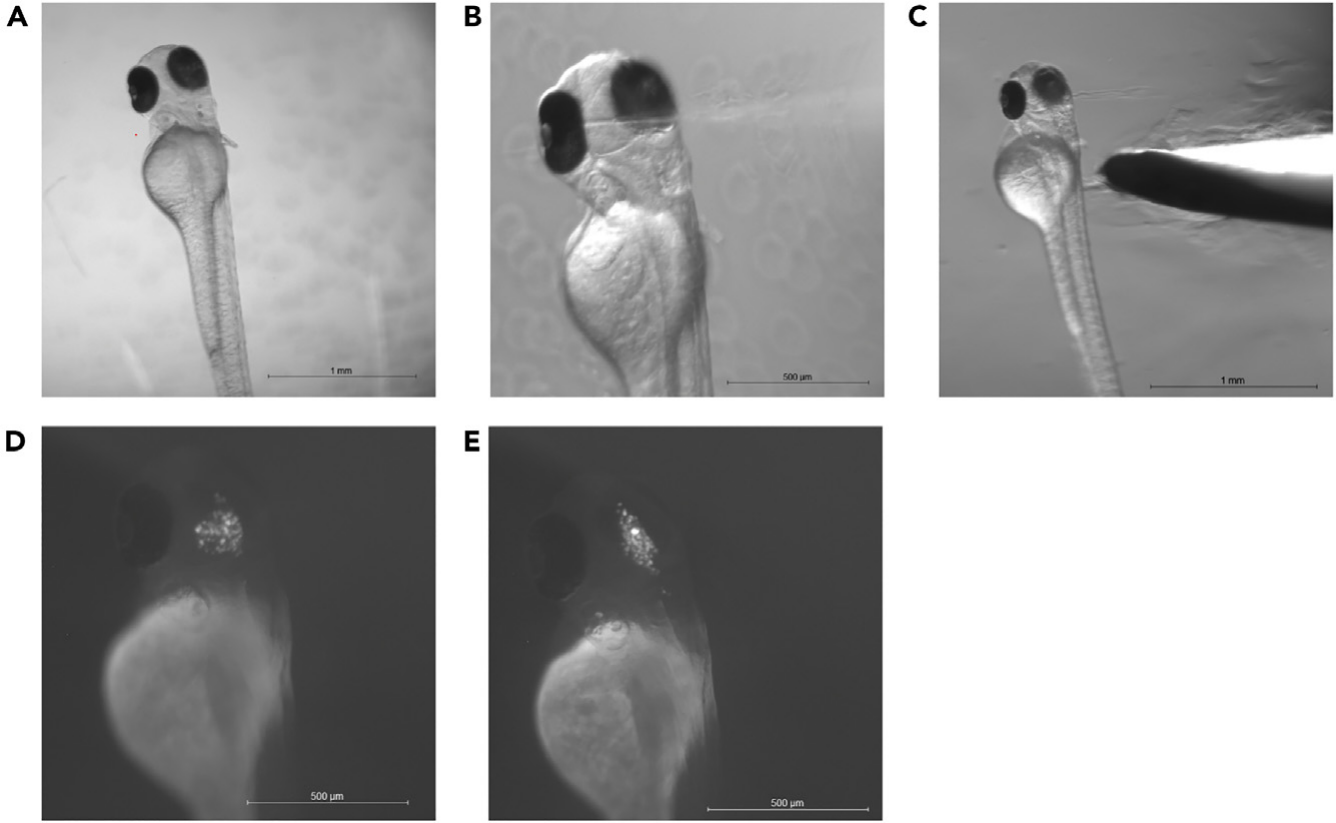
Intracranial cell injection and larva recovery (A) Shows positioning of larva in agarose for intracranial injection. Note the slight lateral tilt towards the injection needle to the right. (B) Shows the insertion of the injection needle to the left hemisphere of the midbrain. In C, the larva is removed from the agarose by scraping the agarose away at the height of the yolk. (D and E) Show fluorescently labeled cells in the midbrain (D) and the midbrain ventricle (E). Scale bar A-C = 1000 μm and D-E = 500 μm.
  b. Position the needle above the left hemisphere of the midbrain, lateral to the ventricle by using a combination of moving the microinjector needle and the injection dish (Figure 6B).
  c. Slowly and carefully pierce the needle through the skin into the midbrain and inject the cells (Video S2; troubleshooting problems 1 and 2).
    ***Note:*** For 12a, in our hands the injections work best when the is positioned with a slight lateral tilt with the brain towards the injection needle (Figures 5D and 6A). The insertion of the needle will cause depression of the skin. Once the skin is pierced through, retract the needle slightly to avoid injection into the ventricle. Adjust the microscope light base contrast settings (f.i. Rottermann Contrast, dark field) to increase the contrast to be able to better evaluate the location of the brain within the larva.
    Video S2. Segmentation of hs-nucleoli positive nuclei, related to steps 36–38 Example movie for the segmentation of human nuclei positive for hs-nucleoli antibody within one z-stack of one embryo. Human and zebrafish nuclei are labeled with DAPI (cyan), hs-nucleoli antibody is shown in magenta. Positive nuclei are outlined in magenta.
  d. Inject all mounted larvae according to steps 9b and 9c and proceed to post-injection procedures (troubleshooting problem 3).

12. Inject the cells into the midbrain of the zebrafish larvae.
  a. Position the embedded larvae under the microscope (Figure 6A).

### Larvae recovery procedures

**Timing: 3 days**

In this step, the removal of the larvae from the agarose and subsequent procedures including incubation, screening of larvae and fixation will be described. Depending on the purpose, the experiments may exceed the larval age of 120 h post-fertilization, for which a license needs to be obtained before starting the experiments.

13. After the injection procedure, remove larvae from the agarose using sharp Dumont forceps or utensils (Figures 5A and 6C).
  **CRITICAL:** Remove all agarose prior to transfer to avoid damage of the larvae.
  ***Note:*** Make sure not to touch the larva while removing it from the agarose. Start scraping an outline around the larva starting at the height of the yolk and subsequently peel off the remaining agarose.
  a. Aspirate the larvae with a 1.5 mL pipette and transfer to a petri dish containing fresh E3 with methylene blue.
  b. Select successfully transplanted larvae using a fluorescent microscope to identify the location of the transplanted cells (Figures 6D and 6E; troubleshooting problem 3).
  c. Place larvae in an incubator at 34°C for 2–3 days, or desired length.
  d. Larvae can be screened for health at each day of incubation and subsequently placed back in the incubator until the desired age is achieved (troubleshooting problem 4).
    ***Note:*** 34°C is the chosen temperature to ensure both cell and larval survival.^10^ When growing zebrafish larvae past 120 hpf, ethical considerations apply, and larvae need to be provided with food and medium changes daily.
14. Select larvae for immunofluorescent staining.
  a. Discard dead as well as visibly damaged and malformed larvae (i.e., tail curled/bended, edema in the heart sack, swelling of head/eyes). Also dispose of larvae where you cannot see fluorescently labeled cells (GSCs) or where the cells are clearly not within the midbrain.
15. Fix the larvae for immunofluorescent staining.
  a. Transfer the larvae to e.g., 1.5 or 2 mL Eppendorf tubes. Remove the remaining E3 medium from the tube and subsequently anesthetize the larvae using 1x Tricaine solution in 1x E3.
  b. Fix the larvae in 4% paraformaldehyde for at least 24 h at 4°C or 3 h at 20°C to preserve the larva and human cells within.

**Pause point:** Larvae can be stored in 1x PBS after fixation for up to 7 days. It is recommended to not exceed this time frame as it might diminish the quality of larvae for further processes.

### Human nucleoli immunofluorescent staining

**Timing: 8–10 days**

In this step, the injected larvae will be subjected to whole mount immunofluorescence staining to visualize human GBM cell nucleoli. This will enable visualization of the human cells (GSCs) during confocal microscope imaging at later time points (troubleshooting problem 5). This protocol describes staining of 120 hpf larvae.

16. Remove the paraformaldehyde in a fume hood and discard the waste according to local regulations. Then, wash the fixed larvae three times for 5 min each using 1x phosphate buffered saline (PBS) in the fume hood.
17. Wash the larvae three times for 5 min using 0.3% PBS-Triton X-100 (PBS-T).
18. Permeabilize for 45 min in 10 μg/mL Proteinase K in PBS at 37°C.

***Note:*** Depending on the age of the larvae the duration of this step can be adjusted. Larvae older than 4 days should be incubated for 45 min, whereas larvae aged 24–48 hpf and larvae aged 48–96 hpf require incubation times of 15 and 30 min respectively.

19. Post-fixate the larvae for 20 min in 4% PFA at 20°C.
20. Wash three times for 5 min in PBS-T.
21. Block with 3% bovine serum albumin (BSA) in PBS-T for 1 h at 20°C.

***Note:*** Dilute the 20% BSA stock to achieve a solution with 3% BSA.

22. Incubate the larvae 12–18 h at 4°C in Eppendorf tubes with the primary antibody (1:100 mouse recombinant anti-human nucleoli antibody) in 3% BSA in PBS-T in a total volume of 100 μL on a rocking platform.
23. Remove the primary antibody by washing the larvae at least five times for 5 min in PBS-T.
24. Block with 3% BSA in PBS-T for 1 h at 20°C.
25. Incubate the larvae 12–18 h at 4°C in Eppendorf tubes with the secondary antibody (1:500 goat-anti-mouse Alexa Fluor 488) in 3% BSA in PBS-T in a total volume of 100 μL on a rocking platform.
26. The following day, remove the secondary antibody by washing the larvae at least five times for 5 min in PBS-T.
27. Incubate the larvae for 5–7 days in 1:1000 DAPI (1 μg/mL) in PBS at 4°C in the dark.
28. After DAPI incubation, wash the larvae with PBS once.

**Pause point:** Larvae can be stored in 1x PBS at 4°C in the dark after immunofluorescence staining for up to 14 days. It is recommended to not store the larvae longer before proceeding with microscopy, as this might reduce image and staining quality.

### Confocal microscopy

**Timing: 3 h (for around 10 larvae)**

This step describes how to image the larvae using an inverted Leica SP8X confocal microscope to generate images that can be quantified with the Cellpose algorithm. This step can vary depending on the microscope setup.

29. Transfer a 1.5 mL tube of 1.5% low-melting point agarose in E3 to a heating block at 60°C.
30. Mount the larvae onto bottom glass dishes using 1.5% low melting point agarose in 1x E3 medium.

***Note:*** For this, transfer the larvae to the glass dish using a plastic transfer pipet and add a drop of agarose on top of the larva using a plastic transfer pipet. Position the larval midbrain as close as possible to the glass, as the microscope used is an inverted microscope. Once the agarose is dried, cover the embedded larvae with 1x PBS to prevent drying out of the sample.

**Figure 7:**
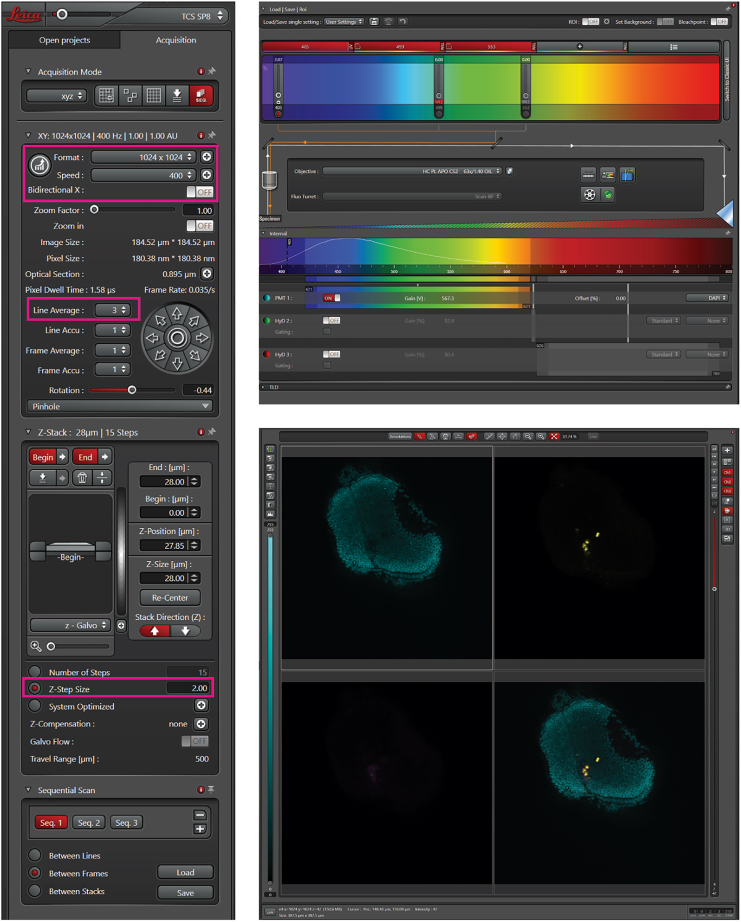
Confocal microscopy setting The left panel shows the overview of standard setting for confocal microscopy. The settings in pink boxes are essential to be able to generate images that can be used for quantification with Cellpose. This includes an image format of 1024 × 1024 with a pixel size of 0.38 μm. The upper right panel shows laser settings to detect cells. The lower right panel shows an overview of example zebrafish brain with zebrafish nuclei in cyan and human cells in yellow.

31. Set up the imaging parameters at the confocal microscope. We typically use the following settings (Figure 7):
  a. 40x oil objective (1.3 NA).
  b. 1024 × 1024 format with a pixel size of 0.38 μm and a zoom factor of 0.75–1.
  c. No bidirectional scanning.
  d. Line average ≥ 3 to reduce noise.
  e. Step size (z) of max. 2 μm (to ensure that Cellpose can detect and segment each individual cell and stitch cells together in a 3D manner).
  f. Laser power as low as possible.
  g. Do not modify any settings between images of one experiment.
  h. Z-stack range adjustable, depending on cell location and working distance of the objective.

**CRITICAL:** The images need to be of good quality for the quantification of human cells. This entails enough resolution throughout the stack as well as bright enough but not oversaturated images.

32. Image the larvae using the settings above and save the imaging file in .lif format.
33. Discard larvae according to institutional regulations.

### Counting nucleoli

**Timing: 2–5 h (depending on dataset)**

**Figure 8:**
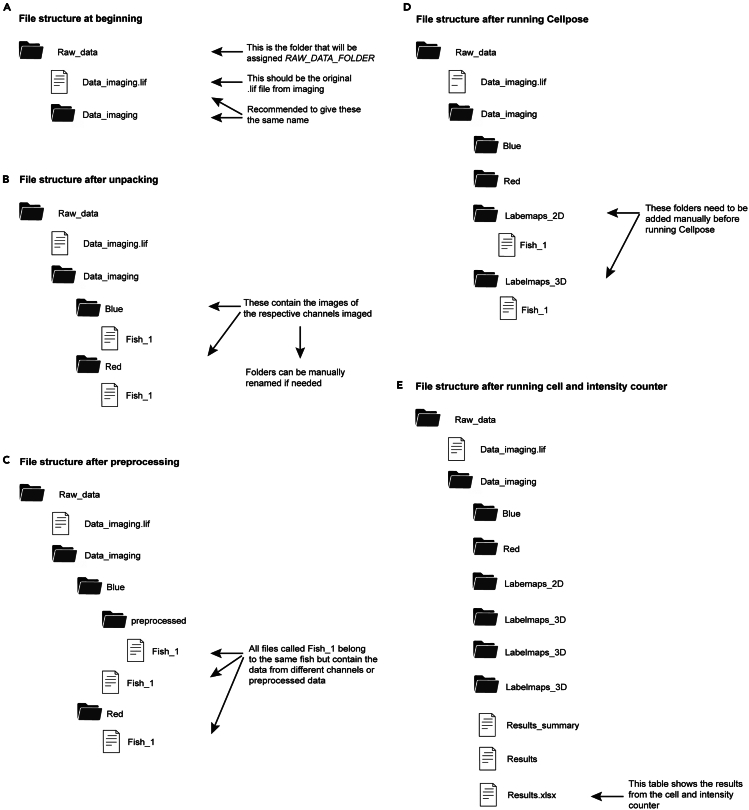
Overview of the file structure when counting nucleoli The figure shows the structure of folders (black icon) and files (white icon) during different steps of the quantification process using the provided code. Panel A shows the structure at the beginning. Panel B shows the structure after unpacking the image files. Panel C shows the structure after preprocessing the unpacked image files. Panel D shows the file structure after running the Cellpose algorithm. Panel E shows the file structure at the end of the section “counting nucleoli”.

In these steps, the algorithm Cellpose and an established Python pipeline will be used to segment and quantify the previously acquired images. More precisely, nuclei that are positive for anti-human nucleoli antibody are detected based on the intensity of the antibody staining. This section includes an explanation for using the Python codes as well as data organization, image preprocessing and cell counting in (parts of) 3D tissue structures using the Cellpose algorithm. We explain the procedures for a Windows-based operating system and for images that are in .lif file format, even though a Mac operating system and other file types can in theory be used as well. Figure 8 shows the file structure during the following steps.

34. Copy your raw data into a “raw data” folder.
  a. Create a folder for raw data in “Documents” and copy the raw imaging data to this folder. It is suggested to call it ‘Raw_Data’ (Figure 8A).

***Note:*** Do not create the raw data folder inside the folder containing the python scripts.

35. If a virtual environment is used, activate it before running any code by typing the following command in the terminal:
  > venv_name\Scripts\Activate.ps1
  ***Note:*** The virtual environment can also be activated by using the select interpreter command from the Command Palette, which can be accessed with Ctrl+Shift+P. Use the zebrafish xenograft cell counter script within the main.py file that was downloaded in 4e to automatically extract images from the raw data .lif file.
  ***Note:*** For the code to fully function, it is important to save all data and required information in the location the code will access. To access the functionality of main.py, copy the lines of code to the interactive window and run them by pressing the ‘**play**’ button or by pressing **shift + enter**. Before using any part of the script, always import the required packages first. These lines of code are usually at the top of the file and start with or contain the word **import**. The package can be imported by running these lines of code in the interactive window.
  a. Open **main.py** and import the required packages.
  b. Add the file path of the folder containing the raw data to the **RAW_DATA_FOLDER** variable in **main.py**.
    **CRITICAL:** Instead of backslashes, use forward slashes (/ instead of \) and have the file path surrounded by quotation marks, for example: RAW_DATA_FOLDER = 'C:/Users/Documents/cellpose_test/experiment_xyz/'
  c. Run the following lines of code:
    > unpack = ImageUnpacker(RAW_DATA_FOLDER)
    > unpack.unpack_images()
    ***Note:***Figure 8B shows the file structure after unpacking. In the case of .lif files, the unpacking module will separate the channels based on the color of the channels that it was given during imaging. Therefore, it will give the folders the names of the colors. These folder names can be changed manually, but make sure to define the new folder names in subsequent steps of the analysis. Sometimes, images from .lif files or other file formats such as .czi or .tif cannot be extracted automatically (see troubleshooting problem 6).
    ***Optional:*** Image size and therefore computing duration can be reduced by manually adjusting the stack size using Fiji. For this, go to *Image>Stacks>Tools>Slice Remover* and indicate which slice should be trimmed from the stack.
36. If necessary, run the automatic preprocessing of the images.
  ***Note:*** In most cases, it is useful to preprocess the nuclear labeling channel (i.e. DAPI channel) and/or the antibody channel to achieve even segmentation by the Cellpose algorithm throughout the 3D stack. This is especially important for bigger z-stacks with clear differences in signal intensity between the most superficial and deeper z-slices, as well as a significant drop in signal quality in the deeper slices. The provided script allows for two different preprocessing options, namely contrast limited adaptive histogram equalization (CLAHE) and standard median filter. It is recommended to run the preprocessing of different channels separately. If multiple parameters should be tested, make sure to save the preprocessed images elsewhere, as otherwise the files will be overwritten once the preprocessing is run again.
  a. Provide the folder names of the channels that need to be preprocessed as a Python list in the variable called: **CHANNELS_TO_PREPROCESS**, see example below. It is recommended to preprocess one channel at a time as different channels usually require different settings due to e.g., varying brightness.
    > CHANNELS_TO_PREPROCESS = ['Cyan']
  b. Provide what type of preprocessing to do as a list in the variable called: **PREPROCESSING_STEPS**.
    ***Note:*** Options: ‘clahe_per_slice’, ‘clahe_total’, ‘median’. Only provide those on the list that are necessary.
    > PREPROCESSING_STEPS = ['clahe_per_slice', 'median']
  c. Provide the parameters for the different preprocessing steps in the variables called: CLIP_LIMIT (CLAHE), NBINS (CLAHE), FOOTPRINT (median filter).
    > CLIP_LIMIT = 0.07 # For clahe histogram equalization
    > NBINS = 127 # For clahe histogram equalization
    > FOOTPRINT = np.ones((5,5)) # For median filter
    **CRITICAL:** There is no definite guideline on which parameters to use as this depends heavily on the image properties. This step requires trials with different values to figure out the most suitable ones for preprocessing for each experiment/dataset/image. Look out for consistent brightness throughout the stack, as well as clear contrast between nuclei and background. It is recommended to try and run Cellpose with different preprocessing parameters and choose based on the outcomes which parameter setting is most suitable.
    ***Note:*** CLIP_LIMIT and NBINS are parameters of **CLAHE** (Contrast Limited Adaptive Histogram Equalization) and will help equalize the signal throughout the whole z-stack, while FOOTPRINT (Standard median filter) will help signal equalization in a single z-slice. Adjusting the CLIP_LIMIT will help balance the contrast and prevent noise amplification. The parameter value is recommended to be set within a range from 0.01 up to 10, with increasing values creating more contrast and increasing brightness. Adjusting NBINS will help with contrast adjustment, with higher values for NBINS resulting in sharper contrast compared to lower values. The range for this parameter is between 0–255. FOOTPRINT adjustment will help remove noise by applying a filter over a set region (the parameter value provided) around each pixel, calculating a median pixel value for the region and replacing the central pixel value with the median pixel value. Increasing the footprint value will increase the area considered for median calculation and can provide strong noise reduction, whereas decreasing the footprint will do the opposite. The recommended range is between 1,1 and 10,10.
  d. Run the following code to run the preprocessing. This will result in a preprocessing subfolder for each channel, which contains the pre-processed images.
    > data_preprocessor = DataPreprocessor(RAW_DATA_FOLDER, CHANNELS_TO_PREPROCESS)
    > data_preprocessor.preprocess_images(preprocessing_steps = PREPROCESSING_STEPS, clipLimit=CLIP_LIMIT, nbins=NBINS, footprint=FOOTPRINT)
    ***Note:***Figure 8C shows the file structure after preprocessing.
37. Use Cellpose to segment the nuclear label channel images and create a 3D and 2D segmentation labelmap.
  **CRITICAL:** If the used computer does not have a GPU, it is advised to run Cellpose on Google Colab (https://colab.research.google.com/) or a local university/institutional computational cluster as otherwise the duration for running the Cellpose will be considerably longer. Contact your institute’s IT department for information and to gain access. An example of how to use our own institutional cluster is explained in “Cell Counter”^5^ and can give an idea on how clusters work in general.
  ***Note:*** The 2D labelmap will have all slices of the z-stack segmented separately, while the 3D labelmap accounts for segmentation throughout the whole stack.
  a. In the **main.py** script, run:
    > from cellpose import models
    > from cellpose import utils
    > from cellpose import plot
    > from skimage.util import img_as_uint
  b. Create folders where the labelmaps will be stored within the folder that contains the channel folders (Figure 8D).
  c. Add the file path of the folder containing the preprocessed data, as well as the results for 2D and 3D labelmaps to the **PATH_DATA, PATH_RESULTS_2D** and **PATH_RESULTS_3D** variables in the **main.py** file, respectively. For example:
    > PATH_DATA = 'C:/Users/Documents/cellpose_test/experiment_xyz/Cyan/preprocessed/'
    > PATH_RESULTS_2D = 'C:/Users/Documents/cellpose_test/experiment_xyz/labelmaps_2D/'
    > PATH_RESULTS_3D = 'C:/Users/Documents/cellpose_test/experiment_xyz/labelmaps_3D/'
  d. Run Cellpose on the (preprocessed) nucleus channel using the following code:
    > image_files = os.listdir(PATH_DATA)
    > model = models.Cellpose(gpu=False, model_type='nuclei')
    > channels = [0,0]
    > for file in image_files:
     img = imread(PATH_DATA + file)
     masks, flows, styles, diams = model.eval(img, diameter=25, flow_threshold=0.9, cellprob_threshold=-6, channels=channels, z_axis=0, do_3D=False, stitch_threshold=0.68)
     labelmap_3D = img_as_uint(masks)
     imsave(PATH_RESULTS_3D + file, labelmap_3D)
     masks, flows, styles, diams = model.eval(img, diameter=25, flow_threshold=0.9, cellprob_threshold=-6, channels=channels, z_axis=0, do_3D=False)
     labelmap_2D = img_as_uint(masks)
    imsave(PATH_RESULTS_2D + file, labelmap_2D)
    ***Note:*** In “> model = models.Cellpose(gpu = False, model_type = 'nuclei')”, gpu = False can be set to gpu = True, depending on the gpu availability. In > for file in image_files: parameters might require adjustment, including diameter, flow_threshold, mask_threshold and stitch_threshold (only for 3D segmentation). It is possible to set do_3D to True, in which case the stitch_threshold parameter is not required. However, we found this mode to be slower for our type of data and segmentation quality seems to suffer, which is why we recommend leaving it set to False.
    ***Note:*** Similarly to what is described in the preprocessing, parameters can be adjusted and need to be tested for each experiment. To check the accuracy of the labelmaps, the labelmap can be opened in Fiji. Using MorphoLibJ plugin>Label Images>Set Labelmap, a colorful labelmap can be created using the golden angle colormap. Nuclei ranging through multiple stacks will have the same color. For this, the image file created in the segmentation folder can be opened in Fiji and compared to the nuclei channel of the raw data file to check whether the nuclei segmentation is accurate. Detailed information on how to set the parameters is documented on the Cellpose website (https://cellpose.readthedocs.io/en/latest/settings.html).
    ***Note:*** In step 37d, the model type is defined as nuclei. The algorithm is also able to identify other cellular structures such as cytoplasm. Please refer to https://cellpose.readthedocs.io/en/latest/models.html for more information.
38. Count the number of immunostained cells using pixel intensity values of the antibody staining.
  a. To count the number of nuclei present in the z-stack, provide the names of the folders in which the label maps are located as strings to the following variables in the **main.py** file: **NAME_FOLDER_2D_LABELMAPS, NAME_FOLDER_3D_LABELMAPS**
  b. Run the following code:
    > counter = DataCellCounter(RAW_DATA_FOLDER, NAME_FOLDER_2D_LABELMAPS, NAME_FOLDER_3D_LABELMAPS)
    > counter.analyze_data()
  c. Identify a subset of cells based on pixel intensity values from immunofluorescent staining by defining the following variables in **main.py** by running the following code:
    > PATH_INTENSITY_IMAGES = 'C:/Users/Documents/cellpose_test/experiment_xyz/Magenta/preprocessed/'
    > NAME_RESULTS_FILE = 'results.json'
    > NAME_LABELMAP_FOLDER = 'labels_total'
    > CHANNELS_TO_USE = ['Magenta']
    > MODE = 'max'
    > CHANNEL_THRESHOLDS_MEAN = [30]
    > CHANNEL_THRESHOLDS_MAX = [100]
    > PREPROCESSED = True
    ***Note:*** For **PATH_INTENSITY_IMAGES** provide the path for to folder in which the preprocessed images from the antibody channel are located.
    For **NAME_RESULTS_FILE** provide results.json, unless the name of the .json file in the dataset folder has been changed.
    For **NAME_LABELMAP_FOLDER** provide the name of the folder containing the 3D label maps that were used for counting the cells.
    For **CHANNELS_TO_USE** list the names of folders containing the data needed to get the subset of cells to count, e.g., ‘Red’.
    For **MODE** provide ‘mean’, ‘max’ or ‘mean/max’ depending on whether the mean pixel value, the max pixel value or both should be used as a threshold.
    For **CHANNEL_THRESHOLDS_MEAN** and/or **CHANNELS_THRESHOLDS_MAX** provide the threshold value(s) to use for mean/max pixel value respectively. The order of values corresponds to the order used in **CHANNELS_TO_USE**. These values can differ between datasets.
    For **PREPROCESSED** provide either true or false, depending on whether the preprocessed images should be used for this analysis.
    ***Note:*** It is possible to count a subset of cells based on pixel intensity values, where maximum and/or mean intensity values in the region corresponding to each label in the Cellpose label maps will be extracted. This means only labels detected to have a mean and/or maximum pixel intensity value above the user-specified threshold will be kept and counted. For this, the value input for the **CHANNEL_THRESHOLDS_MEAN** and/or **CHANNELS_THRESHOLDS_MAX** can be adjusted. To identify which value to provide, one can for example check the intensity value of the antibody staining using other imaging software such as Fiji.
  d. Once these parameters are defined, the intensity counter can be run, and results will be added to the results.xlsx file in the dataset folder (Figure 8E).
    > intensity_counter = DataIntensityCounter(RAW_DATA_FOLDER, NAME_RESULTS_FILE, NAME_LABELMAP_FOLDER, CHANNELS_TO_USE, MODE, CHANNEL_THRESHOLDS_MEAN, CHANNEL_THRESHOLDS_MAX, PREPROCESSED)
    > intensity_counter.count_cells()
    > intensity_counter.save_results()
    ***Note:*** If the cell_counter is run multiple times it is important to note that the existing results will be overwritten. To avoid this, rename the existing results file and save it somewhere else. The same is true for the intensity_counter, which is especially important to note as here settings might have to be adjusted to generate accurate results.
    **CRITICAL:** Running the cell_counter and intensity_counter will generate folders with the segmentation which is used for counting. Use these images to double check the accuracy of segmentation e.g. in FIJI.

## Expected outcomes

**Figure 9:**
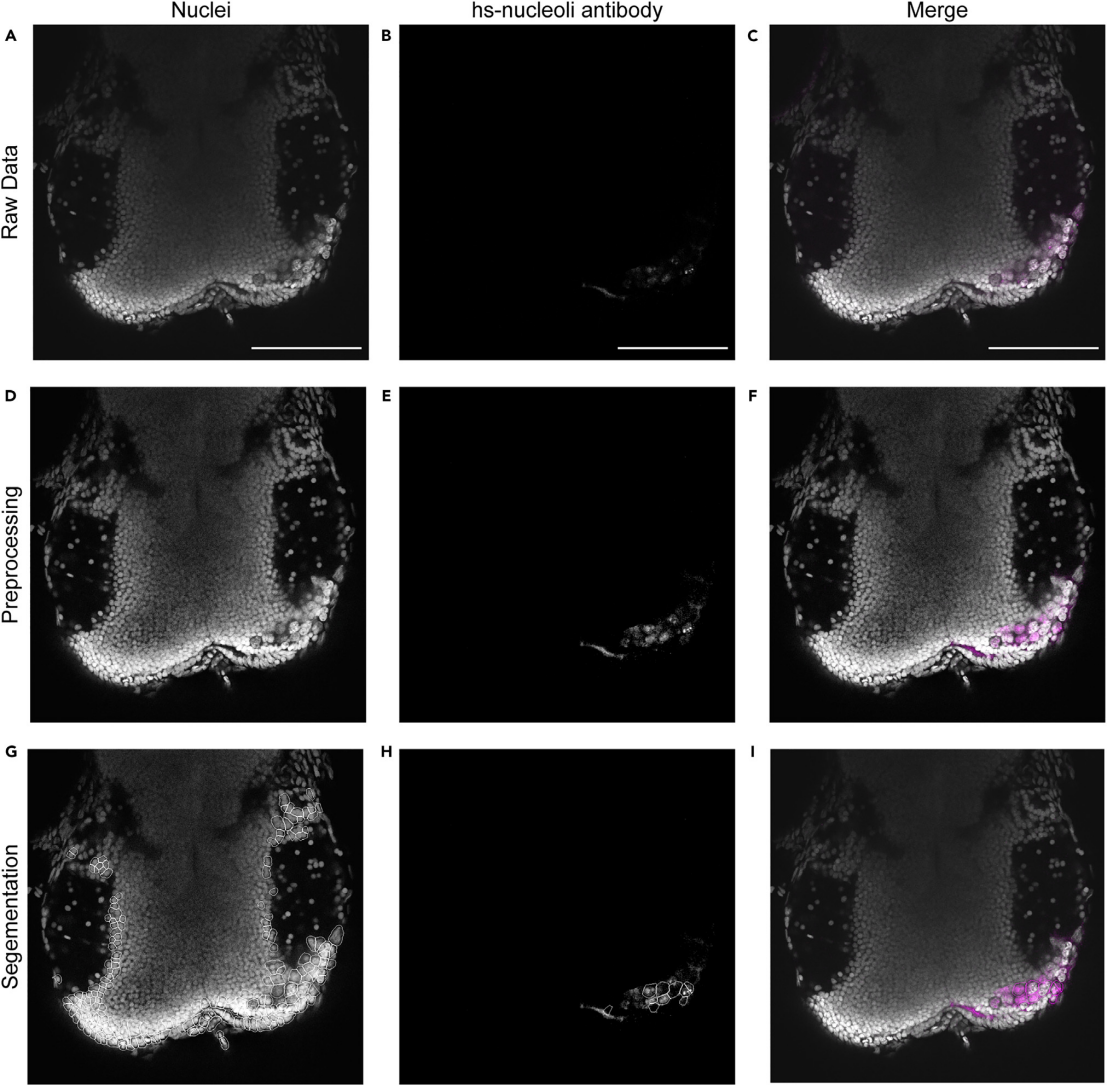
Expected outcomes Shown are different steps for the quantification of human cells using Cellpose. (A–C) Show single slices of raw data of 2 dpf zebrafish midbrain with GG16 cells with extracted channels for nuclei (A), hs-nucleoli antibody (B) and merge of these two channels with nucleoli in magenta (C). (D–F) Show the same images after preprocessing. (G) Shows the segmentation of nuclei used by Cellpose to quantify hs-nucleoli positive nuclei. (H) Shows the segmentation of nuclei with respective hs-nuceloli antibody staining within. (I) Shows the merged image with human nuclei positive for hs-nucleoli antibody outlined in magenta. Scale bar = 100 μm.

**Figure 10:**
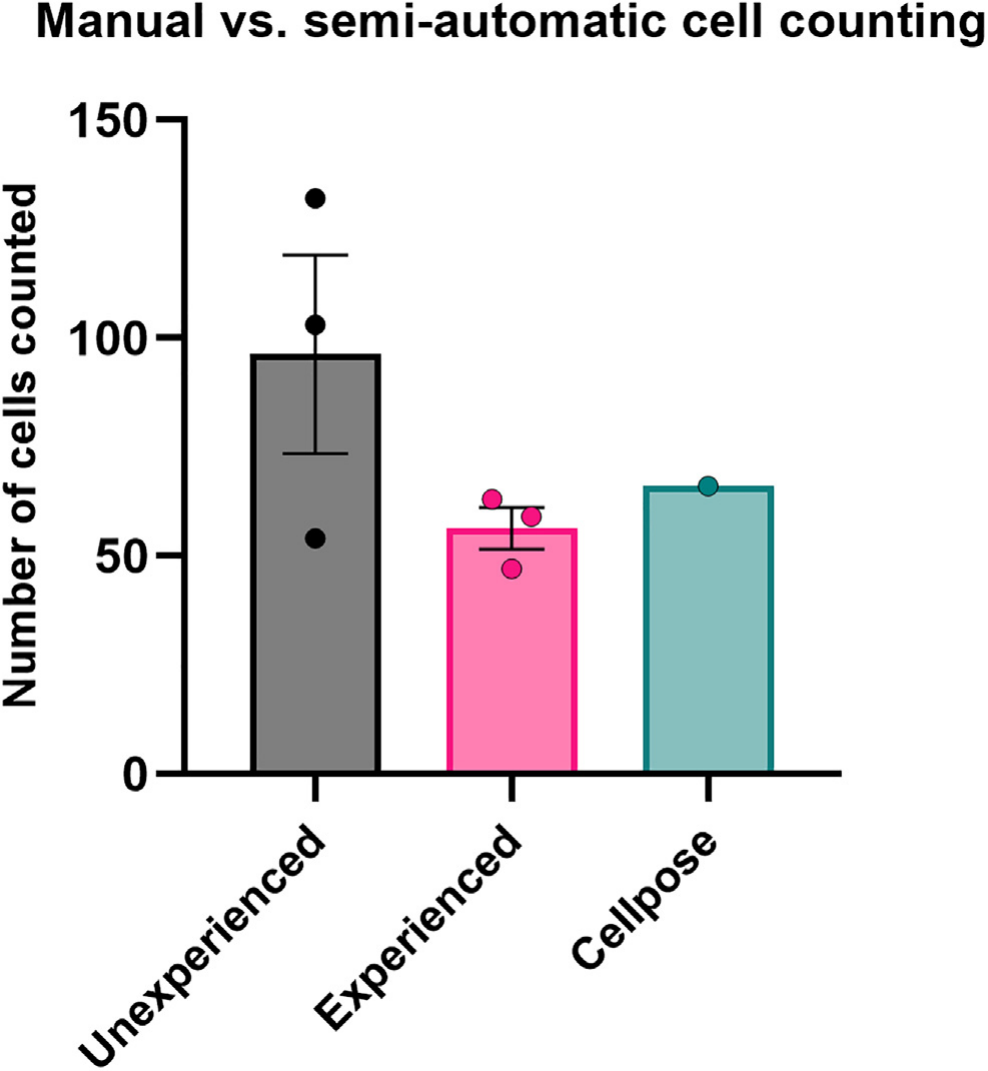
Quantification of human cells Number of GG16 cells present in the zebrafish midbrain for 1 example larva. The number of cells was determined manually, either by researchers unfamiliar with manual cell counting (unexperienced, mean = 96.3, *n* = 3), researchers familiar with manual cell counting (experienced, mean = 56.3, *n* = 3), or determined using the Cellpose pipeline (count = 66, *n* = 1). Data are represented as mean ± SEM. Graph was created using GraphPad Prism.

This protocol provides tools for semi-automatic segmentation and quantification of (human) cancer cells xenografted into zebrafish larvae. Multiple different cell types can be transplanted into different locations of the zebrafish larva (e.g., perivitelline space, midbrain), allowing for a versatile model and comparison between cell types and locations. Once transplanted, larvae are fixed and immunostained and imaged. Based on these images, transplanted cells are preprocessed and subsequently segmented using the Cellpose algorithm (Figure 9, Video S2) and quantified using the provided python script (Figure 10). Manual cell counting of the same sample by unexperienced researchers showed high variability in number of cells counted (96 ± 39), whereas more experienced researchers show lower variability (56 ± 8). Therefore, semi-automatic cell counting provides a more unbiased manner of quantification regardless of experience. With this, cell number and survival upon transplantation can be determined in a reliable and unbiased manner. This method also provides a basis for further analyses and could for example be used in combination with proliferation markers such as the Ki67 antibody. We have not tested further analyses using different antibodies, in principle it is possible to substitute hs-nucleoli antibody with another (human-specific) antibody or using a combination of antibodies with different secondary antibodies. Instead of staining human cells with a fluorescent dye, stably transduced or transfected cancer cells expressing a nuclear localized fluorescent protein (f.i. H2B-mCherry or H2B-eGFP) can be used for quantification. With this, anti-human nucleoli antibody staining could be omitted and xenografted cells with a nuclear signal could be directly quantified using the zebrafish xenograft cell counter.

## Limitations

It is important to realize that raising the zebrafish larvae in elevated temperatures such as described here at 34°C can have effects on zebrafish embryonic development as well as human cell survival. However, 34°C has been described as a temperature where both human cells and zebrafish survive well after xenotransplantation.^11^ Increasing the water temperature during essential stages of embryonic development can lead to mild increases of mortality, as well as malformations.^10^

Further, it is important to note that there are technical limitations of the microscope regarding the imaging depth. For the Cellpose algorithm to be able to identify nuclei, a medium to high spatial resolution and good signal-to-noise ratio is needed, which can only be achieved up to an imaging depth of around 200–300 μm based on our experience. With deeper imaging, the nuclei signal intensity often gets too scattered, leaving Cellpose unable to accurately segment individual cells at this depth. Tissue-clearing techniques or objectives with lower refractive index may be helpful in such instances.^12^

The protocol describes *ex vivo* imaging after immunostaining the larvae, which limits the available information about e.g., cell numbers to a single time point. Here, it is important to note that (human) cell lines expressing a fluorescent protein in the nucleus can be used to avoid antibody staining. It should be possible to use the fluorescent signal generated from these human nuclei for segmentation and quantification, even though this process is not described here. Further, transgenic zebrafish lines can be used to for example visualize zebrafish nuclei (f.i. H2B-eGFP or H2B-mKate) to omit DAPI staining. This could enable live-imaging and therefore the possibility to quantify cancer cells at different time points after xenograft, providing information about cell survival and possible tumor growth. In principle, as long as imaging resolution parameters are upheld, we expect that this protocol can be used to segment transplanted cells using Cellpose based on *in vivo* microscope images.

## Troubleshooting

### Problem 1

Clogging of the needle during cell injection (Step 12c).

### Potential solution

• Unclog needle: Clogging of the needle appears to be mainly caused by formation of cell clumps within the needle. This can be resolved in multiple ways. It is recommended to first try and remove the blockage by pressing the ejection pedal multiple times while the needle is in the medium. If this does not resolve the issue, the ejection pressure can be increased, as well as switching the timed output setting to gated. Be aware that when pressing the pedal in gated mode, the duration of ejection lasts as long as the pedal is pressed, so it can result in ejecting the whole cell suspension. If these options do not resolve the problem, the needle tip can be cut open further using sharp forceps. It is important to note that it should be avoided to open the needle too much as this can result in damage to the brain and often also results in cell injection into the ventricle.
• Replace needle: If all the above solutions do not work, replace the needle with a new one. It is further important to recalibrate the ejection volume after e.g., cutting the needle tip open further. It is recommended to try a few injections into medium after unclogging the needle to make sure the normal cell flow is restored, and the needle does not clog immediately again.

### Problem 2

Difficulties with approaching and piercing through skin when injecting cells (Step 12c).

### Potential solution

• Adjust mounting and needle: When approaching the zebrafish target (in our case the midbrain) with the needle through the agarose, the needle can sometimes move in a more curved than the anticipated straight line. This can be due to excess agarose. To avoid this, during mounting make sure to not use more than a drop of agarose and to position the larva towards the upper part of the agarose drop. Further, when reaching the brain, the skin will depress towards the brain. Carefully advance the needle until you break through the skin. It might be necessary to move the needle upwards again after this to ensure the right location in the midbrain.
• Tap the manipulator: Another option is to carefully tap the back of the manipulator that is holding the needle, thereby causing a small pulse that can penetrate the skin.

### Problem 3

Cells localize in the ventricle instead of the midbrain (Figure 6E) becoming clear due to shape of the cells, as well as cells extending into the fore- and hindbrain (Steps 12c, 13b).

### Potential solution

For this problem, there is not one single solution, as multiple factors can lead to cells ending up in the ventricle.

• Needle shape: Ensure the needle is cut open in a uniform manner, meaning that the needle should have a blunt end. If this is not the case, the needle may appear to be correctly positioned in the brain while it actually is not.
• Injection location: Ensure the injection is not too close to the midline or the mid-hindbrain boundary as this is where the ventricle is located. Injecting too deep in the tissue may result in cells in the ventricle as well.
• Adjust needle angle: Another important aspect is the angle of the injection needle. Based on our experience it is best to try to aim for a 30°–50° angle relative to the surface of the larva head, which will result in the needle entering the brain from dorsal. Further, it is recommended to inject into the hemisphere opposite to the direction of the needle (Figure 6B). Be aware that this technique can be challenging and requires some practice.

### Problem 4

High larval mortality after cell injection (Step 13).

### Potential solution

Potential reasons for larval death include contaminated E3 media, the number of injected cells and volume of injected fluids, too high anesthetic dose or duration during injection and larva recovery from agarose in post-injection procedures. Ensure a clean working space and clean reagents. Further, the injection technique can improve over time and result in less mortality. In principle, larval survival after injection procedure should be ≥90%.

### Problem 5

Anti-human nucleoli antibody signal is weak (Steps 16–28).

### Potential solution

In some cases, the signal of the anti-human nucleoli antibody can be weak.

• Batch effect: In our hands, we have observed batch effects of the antibody, where a certain batch of anti-human nucleoli antibody yielded lower signal intensities compared to another.
• Extend washing: For this, try carefully executing and possibly extending the washing steps (especially after Proteinase K treatment).
• Extend Proteinase K incubation time: Proteinase K incubation time can be extended, but in a careful manner as the tissue can become too permeabilized, i.e., it begins to fall apart.
• Increase antibody concentration: Another solution is to increase the concentration of primary antibody used.

### Problem 6

Images cannot be automatically extracted from raw data using “ImageUnpacker” (Step 35) or are not in .lif file format.

### Potential solution

Images can alternatively be extracted manually by using software such as FIJI. Note that inside the “RAW_DATA_FOLDER” new folders for individual channels must be created manually. Save single-channel z-stacks as .tif files and move them into the according channel folder.

### Problem 7

Error messages arise when running the provided code.

### Potential solution

Unfortunately, we cannot provide a potential solution for every error message that may arise. In case the following examples are not contributing to solving the problem, we recommend searching online for similar error messages, as platforms such as stackoverflow.com, github.com, cellpose.readthedocs.io or image.sc offer a wide variety of solutions.

• FileNotFound: Check whether the file is in the folder the script is trying to access and correct the path or file location.
• Module not found/no module named: Install the required module by running “pip install – name module” in the interactive window. Further, make sure you are in the virtual environment in which the modules have been installed during the setup.
• NameError: This usually means a name is not defined, so re-run the definition of that name in the interactive window.

## Resource availability

### Lead contact

Further information and requests for resources and reagents should be directed to and will be fulfilled by the lead contact, Karina Köpke (k.kopke@umcg.nl).

### Technical contact

Questions about the technical specifics of performing the protocol should be directed to the technical contact, Karina Köpke (k.kopke@umcg.nl).

### Materials availability

This study did not generate new unique reagents.

### Data and code availability

The code generated during this study is available on GitHub (https://github.com/nynkeoosterhof1/xenograft-quantification). An archived version of record is available at https://doi.org/10.5281/zenodo.13910146.

## Acknowledgments

This study was funded by a University Medical Center Groningen – BCN PhD fellowship to K.K. and a Rosalind Franklin Fellowship from the University of Groningen, co-funded by EU/FP7-PEOPLE, and a Dutch Research Council (NWO) Vidi grant (016.Vidi.171.016) to J.T.M.L.P. We thank Daniëlle Koot, Xiao Ling, and Maria F. Suárez Peredo Rodríguez for help with manual cell counting for the quantification section and Marsudi Siburian for test running the section “Counting nucleoli.” We thank the UMCG animal facility for excellent zebrafish care. Part of the work has been performed at the UMCG Microscopy and Imaging Center (UMIC), which is sponsored by the Dutch Research Council (NWO) grant 40-175-010-2009-023. The graphical abstract was created using BioRender.com.

## Author contributions

Conceptualization, K.K. and J.T.M.L.P.; code development, N.O.; methodology, K.K., C.-S.L., and N.O.; experimentation, K.K. and E.S.C.D.; analysis, K.K., C.-S.L., and J.T.M.L.P.; writing – original draft, K.K. and J.T.M.L.P.; writing – reviewing and editing, K.K., C.-S.L., N.O., and J.T.M.L.P.; supervision and funding acquisition, J.T.M.L.P.

## Declaration of interests

The authors declare no competing interests.
